# Nitrogen Level Changes the Interactions between a Native (*Scirpus triqueter*) and an Exotic Species (*Spartina anglica*) in Coastal China

**DOI:** 10.1371/journal.pone.0025629

**Published:** 2011-10-05

**Authors:** Hong-li Li, Guang-chun Lei, Ying-biao Zhi, Shu-qing An, He-ping Huang, Yan Ouyang, Lei Zhao, Zi-fa Deng, Yu-hong Liu

**Affiliations:** 1 College of Natural Conservation, Beijing Forestry University, Beijing, China; 2 The State Key Laboratory of Pollution Control and Resource Reuse, School of Life Science, Nanjing University, Nanjing, China; 3 The Institute of Wetland Ecology, School of Life Science, Nanjing University, Nanjing, China; 4 School of Tourism and Urban Management, Jiangxi University of Finance and Economics, Nanchang, China; 5 Yantai Institute of Coastal Zone Research for Sustainable Development, CAS, Yantai, China; Purdue University, United States of America

## Abstract

The exotic species *Spartina anglica*, introduced from Europe in 1963, has been experiencing a decline in the past decade in coastal China, but the reasons for the decline are still not clear. It is hypothesized that competition with the native species *Scirpus triqueter* may have played an important role in the decline due to niche overlap in the field. We measured biomass, leaf number and area, asexual reproduction and relative neighborhood effect (RNE) of the two species in both monoculture and mixture under three nitrogen levels (control, low and high). *S. anglica* showed significantly lower biomass accumulation, leaf number and asexual reproduction in mixture than in monoculture. The inter- and intra-specific RNE of *S. anglica* were all positive, and the inter-specific RNE was significantly higher than the intra-specific RNE in the control. For *S. triqueter*, inter- and intra-specific RNE were negative at the high nitrogen level but positive in the control and at the low nitrogen level. This indicates that *S. triqueter* exerted an asymmetric competitive advantage over *S. anglica* in the control and low nitrogen conditions; however, *S. anglica* facilitated growth of *S. triqueter* in high nitrogen conditions. Nitrogen level changed the interactions between the two species because *S. triqueter* better tolerated low nitrogen. Since *S. anglica* is increasingly confined to upper, more nitrogen-limited marsh areas in coastal China, increased competition from *S. triqueter* may help explain its decline.

## Introduction

Salt marsh communities are often characterized by elevational gradients [1], [2]. Such patterns often result from trade-offs between plant competitive ability and tolerance of stresses such as flooding, salinity and low nitrogen [3]–[6]. Interactions between plants can strongly affect community structure and can also be changed by environmental factors [7]–[14].

Nutrients, especially nitrogen, are an important factor that may affect plant interactions and succession of salt marsh communities [10], [12], [15], [16]. Nitrogen addition was found to change the relative abundance of *Spartina foliosa* and *Salicornia bigelovii* in a California salt marsh [17]. The interactions between *Spartina anglica* and *Puccinella maritima* were facilitative in low nutrient conditions, but not in high nutrient conditions [4]. The ericoid species *Calluna vulgaris* and *Vaccinium oxycoccus* were stronger competitors than the graminoid species *Eriophorum vaginatum* and *Rhynchospora alba* at high nutrient levels in peat bogs, but not at low nutrient conditions [18].

*Spartina anglica* is a perennial salt marsh grass native in England [19], [20]. Invasions of *S. anglica* in other countries or regions have caused great changes in local communities [21], [22]. *S. anglica* was first introduced in coastal China in 1963 [23], [24], and grew to cover 36,000 ha by 1985 [25], [26]. In the past decade, however, large-scale decline of the species has been occurring in coastal China, and the cover has decreased to less than 50 ha [26], [27].

Due to the wide spread of *Spartina alterniflora* in coastal China [23], the ecological range of *S. anglica* is restricted to higher elevations where the native, rhizomatous species *Scirpus triqueter* is abundant. Therefore, *S. anglica* and *S. triqueter* currently possess overlapping ecological niches in the intertidal zone in China, and may compete strongly for space, nutrients and light.

In this study, we aim to test the hypothesis that competition between *S. anglica* and *S. triqueter* is one possible explanation for the on-going decline of *S. anglica* in coastal China. Because interactions between species, especially between exotic and native species, often depend on environmental conditions [6], [28], and because nitrogen is one of the most important environment factors that limit the growth of salt marsh plants [17], [29], we also test whether nitrogen addition affects the interactions between *S. anglica* and *S. triqueter* and whether the results can help explain the decline of *S. anglica* in coastal China.

## Materials and Methods

### The species

*Spartina anglica* C. E Hubbard (cordgrass) is a rhizomatous perennial grass that spreads mainly by clonal growth [30]. The flowers occur in numerous, erect, dense panicles that consist of closely overlapping spikelets in two rows on one side of the rachis [31]. In Europe the flowers produce viable seeds through both self- and cross-pollination that is mainly by wind. However, seed production has changed significantly over years, especially in China [25]. Viable seed production has diminished [26] due to poor pollen quality and abnormal pollen tubes [32]. The height of the plant has decreased from 100 cm to not more than 30 cm in coastal China.

*Scirpus triqueter* Linn. is trigonous stems and about 100 cm tall, with leafless sheaths below. The uppermost sheaths usually have a short lamina, and the glumes are between 3.4 and 4 mm. *S. triqueter* is characterized by two stigmas and nuts between 2.5 and 3 mm [33]. It occurs in different habitats in tidal wetlands that range from brackish to fresh water along the coast.

### Plant materials

In April 2007, plants of *S. anglica* and *S. triqueter* were collected from the same area of the marsh zone (120°15′E and 33°42′N) at Xinyang Harbor in Yancheng Nature Reserve in Jiangsu Province, China. The plants were carefully collected from the marsh and transplanted into big trays (length 75 cm, width 52 cm and height 41 cm) filled with a 30-cm-deep 1∶1 (v∶v) mixture of sand and clay under greenhouse conditions. For each species, individual plants consisting of a single tiller with attached roots were separated from the plants in the trays. Uniformly sized ramets with a height of 12.6±0.5 cm (for *S. anglica*) and 15.2±0.5 cm (for *S. triqueter*) were selected and planted into pots (10 L in volume, 28 cm in diameter, 20 cm in height) containing the same soil mixture of 15 cm in depth. Biomass of the two species was 0.267±0.008 g and 0.283±0.005 g, respectively, at the beginning of the experiment. The plants were allowed to acclimatize for six days before experimental treatments began in May 2007.

### Experimental design

The experiment took a randomized block design, with nitrogen level and species combination as the main factors. There were five species combination treatments, i.e., each pot (28 cm in diameter) was planted with (i) only 2 individuals of *S. anglica* (coded as “SA2”), (ii) only 2 individuals of *S. triqueter* (ST2), (iii) 4 individuals of *S. anglica* (SA4), (iv) 4 individuals of *S. triqueter* (ST4), and (v) 2 individuals of *S. anglica* and 2 individuals of *S. triqueter* (SA2+ST2). When the pot was planted with two individuals (treatments of SA2 and ST2), the two individuals were spaced 7.3 cm apart along a diameter of the pot. When the pot was planted with four individuals (treatments SA4, ST4 and SA2+ST2); two individuals were located along one diameter with 7.3 cm apart, and the other two were planted along its perpendicular diameter also with 7.3 cm apart. For SA2+ST2, the two individuals of the same species were planted along the same diameter. There were three nitrogen treatments, i.e., control (no added N), low nitrogen, and high nitrogen, imposed by hand-broadcasting a total of 0, 2.5 and 5.0 g of fast-release, (NH_2_)_2_ CO pelletized fertilizer, respectively, over the surface of the soil in each pot every three months from May to December 2007. In total there were 15 treatments and each treatment had four replicates (pots) arranged in four different blocks.

The plants for each treatment were selected at random from the experimental stock in an attempt to reduce the possible influence of clonal variation and plant history. Pots were randomly located and rotated every other week. Salinity was maintained at about 15.8 ppt. Water level was maintained at the level of the soil surface. We weeded the experimental pots for the first 24 weeks, after which no more weeds appeared.

### Data collection

Plants were harvested during 1 to 6 December 2007. The pots were upended to remove plants, and the plants were then rinsed in fresh water. For each species in each pot, leaf area and number of leaves were used as measures of leaf performance, and total length of rhizomes and number of ramets as measures of asexual performance. Leaf area was measured using a Licor-3000 electronic conveyor. Plants were then divided into leaves and stems (i.e., shoots), rhizomes and roots, dried to constant mass at 80°C, and weighed. No plants flowered during the experiment. The average values for each parameter were calculated for each pot.

### Data analysis

Statistical analyses were conducted in SPSS 17.0 for Windows (SPSS Inc., USA). We used three-way ANOVAs to test the effect of block, nitrogen, species combination and nitrogen by species combination on the variables measured. *P*<0.05 was considered to be statistically significant. Measures of biomass were transformed to the log as needed to improve homogeneity of variance prior to ANOVA.

The intensity of inter- and intra-specific interactions between the two species was quantified using relative neighborhood effect [4], :
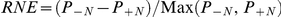
where RNE is relative neighborhood effect, *P_−N_* and *P_+N_* are a measure of plant performance in the absence or presence of intra- or inter-specific neighbors, respectively, and Max (*P_−N_*, *P_+N_*) is the larger value of *P_−N_* and *P_+N_*. RNE varies from −1 to 1; a value of 0 indicates no interaction, with negative values indicating facilitation and positive values indicating competition.

The different combinations of the two species tested the presence or absence of neighbors; treatments with two plants of the same species per pot tested growth in the absence of neighbors, while those with four plants of the same species per pot or with two of each species, respectively, tested growth in the presence of intra-specific or inter-specific neighbors. The intensity of intra- and inter-specific interactions was determined using total biomass as an indicator of plant performance. A Duncan test was conducted to examine the difference in intra- and inter-specific interactions among the treatments.

## Results

### Biomass

**Figure 1:**
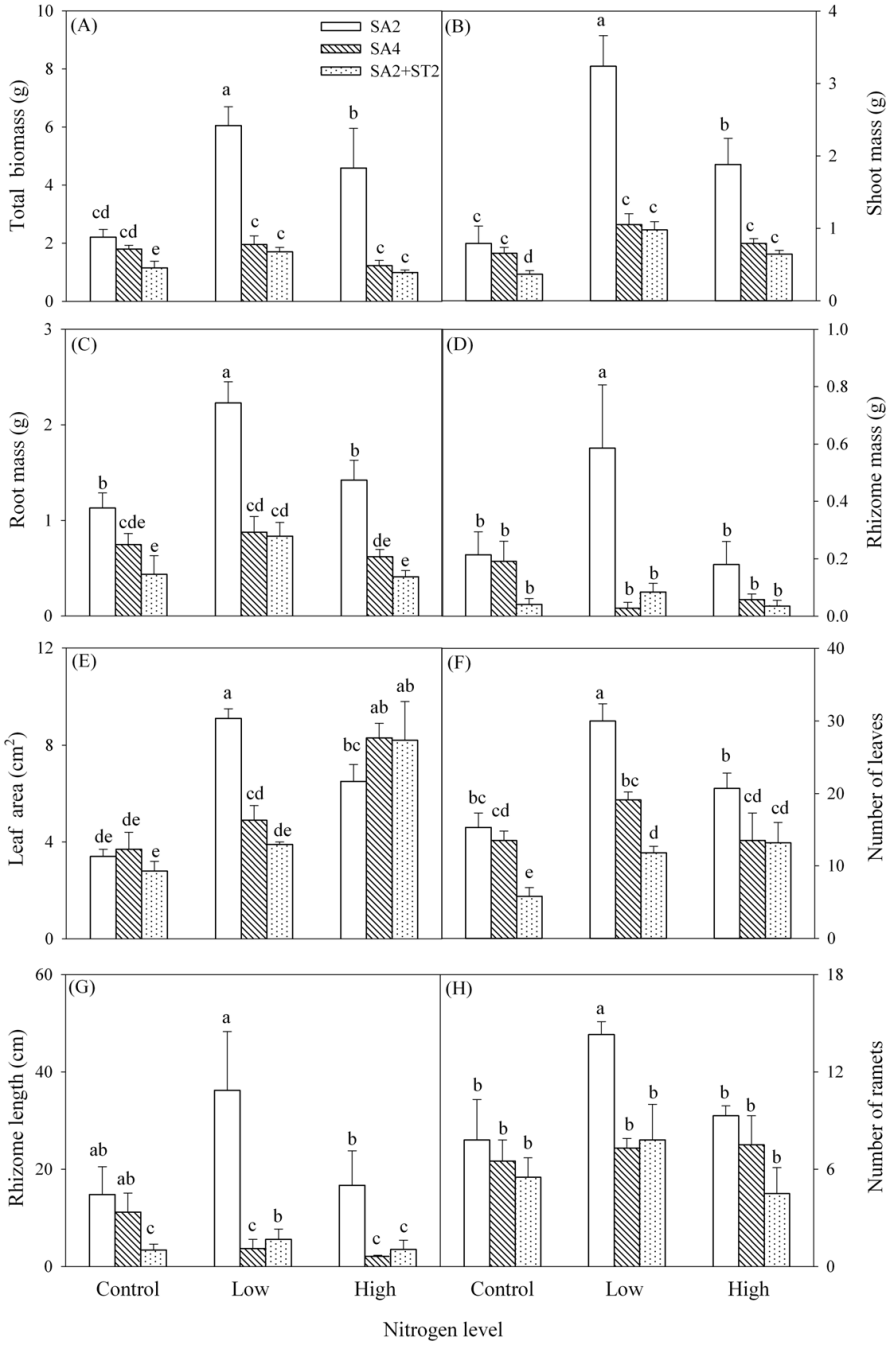
Biomass, leaf and asexual characteristics of *S. anglica* in nitrogen level and species combination treatments. Different lowercase letters indicate that groups differ significantly.

Total biomass, shoot mass and root mass of *S. anglica* in SA2 were significantly higher in the low nitrogen treatment than in the high nitrogen treatment (Fig. 1A–C; Table 1A). Rhizome mass in SA2 was significantly greater in the low nitrogen treatment than in the control and the high nitrogen treatment (Fig. 1D; Table 1A). In the control, total biomass and shoot mass of *S. anglica* were significantly less in the two-species treatment (SA2+ST2) than in the single-species treatments (SA2, SA4) (Fig. 1A,B).

**Table 1:** Effects of block, nitrogen level and species combination on growth and asexual reproduction of *Spartina anglica* (A) and *Scirpus triqueter* (B).

Variable	Block	Nitrogen (N)	Combination (C)	N×C
(A) *Spartina anglica*				
Total biomass	0.4 ^ns^	24.1^**^	47.5^**^	9.8^**^
Shoot mass	0.8 ^ns^	21.1^**^	31.5^**^	6.9^**^
Root mass	1.2 ^ns^	13.8^**^	45.0^**^	3.9^*^
Rhizome mass	0.2 ^ns^	1.9 ^ns^	7.6^**^	2.8^*^
Leaf area	0.4 ^ns^	29.6^**^	2.9 ^ns^	7.6^**^
Number of leaves	1.1 ^ns^	12.5^**^	18.4^**^	4.7^*^
Rhizome length	1.4 ^ns^	1.7^ns^	9.5^**^	2.0^ns^
Number of ramets	2.4 ^ns^	4.2 ^*^	7.8^**^	1.4^ns^
(B) *Scirpus triqueter*				
Total biomass	3.0^ns^	37.7^**^	0.2^ns^	3.4^*^
Shoot mass	2.1 ^ns^	37.2^**^	0.01^ns^	2.6^ns^
Root mass	2.3 ^ns^	22.6^**^	0.3 ^ns^	2.0^ns^
Rhizome mass	0.9 ^ns^	0.04^ns^	1.2^ns^	2.5 ^ns^
Leaf area	0.5 ^ns^	139.1^**^	31.5^**^	4.4^**^
Number of leaves	1.2 ^ns^	35.3^**^	5.6^*^	3.8^*^
Rhizome length	0.4 ^ns^	3.8^*^	3.3 ^ns^	0.6^ns^
Number of ramets	1.3 ^ns^	26.8^**^	2.3^ns^	0.2^ns^

*F*- values and significance levels (^**^ *P*<0.01, ^*^ *P*<0.05, ^ns^ *P*≥0.05) are given. Degrees of freedom for the block effect, nitrogen level, species combination and interaction between nitrogen and combination are (3, 24), (2, 24), (2, 24) and (4, 24), respectively.

**Figure 2:**
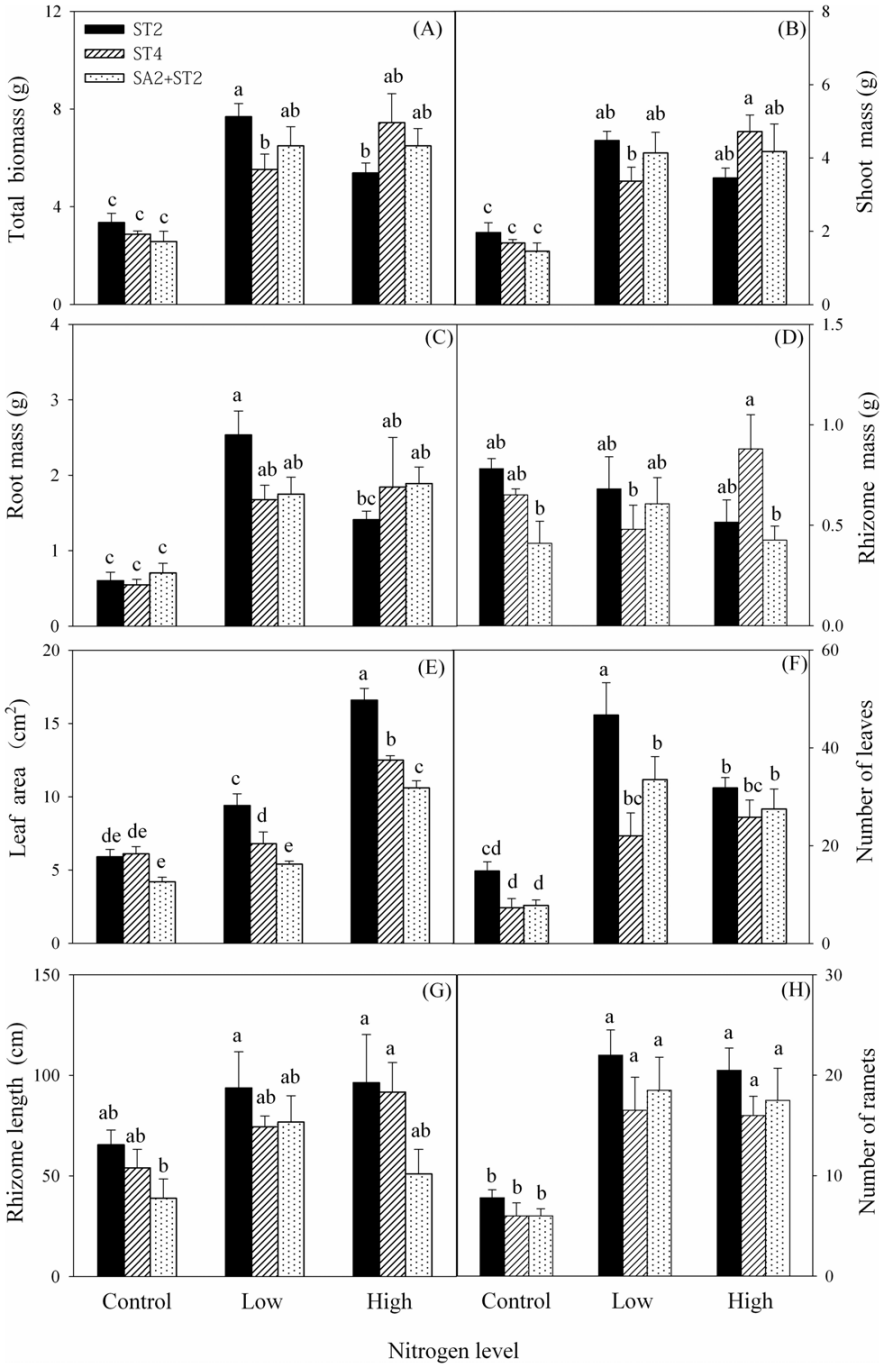
Biomass, leaf and asexual characteristics of *S. triqueter* in nitrogen level and species combination treatments. Different lowercase letters indicate that groups differ significantly.

Total biomass, shoot mass and root mass of *S. triqueter* were greater in the low and the high nitrogen treatment than in the control (Fig. 2A–C; Table 1B). Biomass measures of *S. triqueter* did not differ significantly among ST2, ST4 and SA2+ST2 in the control or the high nitrogen treatment, except for rhizome mass in the high nitrogen treatment and total biomass in the low nitrogen treatment (Fig. 2 A–D).

### Leaf area and number

Leaf area of *S. anglica* was greater in the low and the high nitrogen treatment than in the control (Fig. 1E; Table 1A). Leaf area of *S. anglica* was significantly greater in SA2 than in SA4 and SA2+ST2 in the low nitrogen treatment, but did not differ among SA2, SA4 or SA2+ST2 in the control or the high nitrogen treatment (Fig. 1E). Number of leaves was smaller in SA2+ST2 than in SA2 and SA4 in the control and the low nitrogen treatment (Fig. 1F; Table 1A).

In *S. triqueter*, leaf area and number were greater in the low and the high nitrogen treatment than in the control (Fig. 2E, F; Table 1B). In the low and the high nitrogen treatment, leaf area was the largest in ST2, smallest in SA2+ST2, and intermediate in ST4; in the control, it did not differ among ST2, ST4 or SA2+ST2 (Fig. 2E). Number of leaves was significantly larger in ST2 than in ST4 and SA2+ST4 in the low nitrogen treatment, but did not differ among them in the control or the high nitrogen treatment (Fig. 2F).

### Asexual reproduction

Total rhizome length of *S. anglica* was significantly greater in SA2 than in SA4 and SA2+ST2 in the low and the high nitrogen treatment, and was smaller in SA2+ST2 than in SA2 and SA4 in the control (Fig. 1G; Table 1A). In the low nitrogen treatment, number of ramets of *S. anglica* was significantly greater in SA2 than in SA4 and SA2+ST2 (Fig. 1H).

In *S. triqueter*, number of ramets was significantly greater in the low and high nitrogen treatment than in the control (Fig. 2H; Table 1B). But total rhizome length did not differ significantly among the three nitrogen treatments (Fig. 2G; Table 1B).

### Relative neighborhood effect

**Figure 3:**
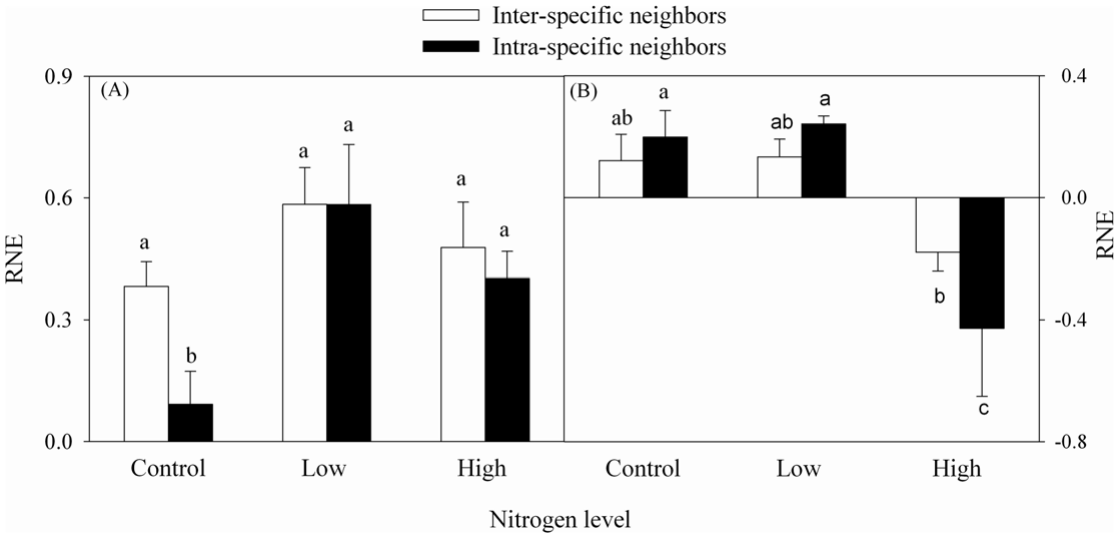
Inter-specific and intra-specific relative neighborhood effect (RNE) of *S. anglica* (A) and *S. triqueter* (B). Different lowercase letters indicate that groups differ significantly.

In *S. anglica*, the values of RNE were all positive (Fig. 3A), suggesting that the effects of neighbor plants on *S. anglica* were all competitive. In *S. anglica*, RNE was significantly higher when the neighbors were a different species (inter-specific RNE) than when the neighbors were the same species (intra-specific RNE) in the control, but they did not differ significantly in the low or the high nitrogen treatment (Fig. 3A).

In *S. triqueter*, the values of RNE were positive in the control and the low nitrogen treatment, but became negative in the high nitrogen treatment (Fig. 3B), suggesting that the effects of neighbors on *S. triqueter* changed from competition to facilitation with increasing the nitrogen levels. The inter-specific RNE did not differ significantly from the intra-specific RNE in the control or the low nitrogen treatment, but was significantly larger than the intra-specific RNE in the high nitrogen treatment (Fig. 3B).

## Discussion

Values of RNE indicate that inter-specific competition was more intense than intra-specific competition, and that *S. triqueter* was competitively dominant over *S. anglica*. Competition between the two salt marsh species in the field is likely because their niche overlaps [23]. However, the two species differed substantially when facing interference competition. When grown in monoculture without added nitrogen, biomass, leaf area and asexual reproduction of both species were slightly reduced in the presence of neighbors (Figs. 1 and 2). This is consistent with previous research [3], [4], [27]. Differences between the inter-specific and the intra-specific RNE of the two species (Fig. 3) indicate that *S. triqueter* was less affected by *S. anglica* than by *S. triqueter*, whereas *S. anglica* was more affected by *S. triqueter* than by *S. anglica*. Thus, *S. triqueter* has a stronger inter-specific competitive ability and a weaker intra-specific competitive ability than *S. anglica*.

Many studies have shown that environmental conditions can change inter-specific interactions [3], [4], [6], [18], [34]–[36]. Our study also showed that nitrogen level changed the interactions between the two species. The effects of *S. triqueter* on biomass and asexual reproduction of *S. anglica* changed with increasing nitrogen levels. Without nitrogen addition (in the control), inter-specific competitive ability was lower than intra-specific competitive ability in *S. triqueter*; in contrast, *S. anglica* had higher inter-specific competitive ability and low intra-specific competitive ability. These results suggest that *S. anglica* was strongly affected by *S. triqueter*, but *S. triqueter* was not strongly affected by *S. anglica*. Thus, without nitrogen addition *S. triqueter* was competitively dominant over *S. anglica*. At the low nitrogen level, patterns in competitive ability were similar to those in the control. At the high nitrogen level, however, the interactions between the two species changed greatly: in *S. triqueter*, inter- and intra-specific interactions became facilitative rather than competitive, whereas in *S. anglica* only inter-specific interactions were important (Fig. 3). Thus, the interactions between *S. anglica* and *S. triqueter* were determined to some extent by abiotic factors, as found in other research [34]–[37].

Competition for resources is mainly due to competition for light and nutrients [38]–[40]. Our results indicate that *S. triqueter* had a higher and denser canopy than *S. anglica* and could thus shade *S. anglica* to some extent, consistent with previous findings [41]–[43]. In the case of competition for nutrients, the relative allocation of biomass to shoots and roots provides insight into the competitive mechanisms employed by different species [39]. In the control and the low nitrogen level, *S. triqueter* responded to competition by increasing shoot growth, and thus produced a dense, highly branched canopy; in the high nitrogen level, its growth was faciliated by neighbors. This suggests that *S. triqueter* has a higher tolerance for low nitrogen availability than *S. anglica*.

The inter-specific competitive advantage of *S. triqueter* shown in this study may explain the decline of *S. anglica* in coastal China. When *S. anglica* was first introduced to China, it occupied the open coastal areas and expanded its population quickly [23], [26]. The conditions had changed because *S. alterniflora* had accelerated natural land formation by expanding over tremendous areas with a vertical accretion rate of 48–52 cm in 3–4 years [24]. Because of this vertical accretion, the hypsography is higher than that of 30 years ago and only spring tides can reach the current population of *S. anglica* in the upper marshes where *S. triqueter* is abundant. Due to accretion, these areas have also become poor in nitrogen, which may limit the growth of *S. anglica*. The fact that *S. triqueter* is more tolerant to low nitrogen than *S. anglica* can further explain the decline of *S. anglica* in coastal China.
